# SELF-COMPASSION IN CAREGIVERS OF NEURODEGENERATIVE DISEASES: DEMENTIA CARE CONTEXTS AND BEYOND

**DOI:** 10.1093/geroni/igae098.2828

**Published:** 2024-12-31

**Authors:** Christi Lero, Soobin Park, Emily Mroz

**Affiliations:** Washington University in St. Louis, St. Louis, Missouri, United States; Washington University in St. Louis, St. Louis, Missouri, United States; Emory University, Decatur, Georgia, United States

## Abstract

Informal caregivers of those with neurodegenerative disease (ND) manage increasingly complex symptoms which impact their own wellbeing. Self-compassion can promote maintenance of wellbeing during challenging life experiences, including caregiving. There is little guidance for studying self-compassion observationally or targeting self-compassion through intervention for this population. Our objective was to complete a scoping review of research describing self-compassion in the context of wellbeing of informal caregivers of those living with ND. In this review, following PRISMA-ScR guidelines, three online databases identified 350 peer-reviewed articles, 18 of which were included in this study. Eligibility included being written in English, targeting informal caregivers of a care recipient living with ND, and examination of self-compassion. Findings showed that study samples most often included informal caregivers of persons living with Alzheimer’s and related dementias. Across study types (e.g., hypothesis generating, intervention) self-compassion appeared as a theoretical concept, emerging theme from participant interviews, variable associated with other outcomes, and a main outcome variable. Self-compassion is often measured using the Self-Compassion Scale or short form version of this measure. Based on this review, we will discuss how study of self-compassion as useful for informal caregivers of individuals living with ND is in a growth period. The classification of ND is broad, however the range of common NDs among care recipient diagnosis was not represented by existing work. Future research should explore the nuances of self-compassion when caregiving for persons living with other NDs, as symptomology, disease trajectory, treatments, care provision, and outcomes vary across these caregiving contexts.

